# Neutrophil Cathepsin G, but Not Elastase, Induces Aggregation of MCF-7 Mammary Carcinoma Cells by a Protease Activity-Dependent Cell-Oriented Mechanism

**DOI:** 10.1155/2014/971409

**Published:** 2014-04-02

**Authors:** Satoru Yui, Yuuki Osawa, Takeo Ichisugi, Riyo Morimoto-Kamata

**Affiliations:** Laboratory of Host Defense, Department of Pharma-Sciences, Teikyo University, 2-11-1 Kaga, Itabashi-ku, Tokyo 173-8605, Japan

## Abstract

We previously found that a neutrophil serine protease, cathepsin G, weakens adherence to culture substrates and induces E-cadherin-dependent aggregation of MCF-7 human breast cancer cells through its protease activity. In this study, we examined whether aggregation is caused by degradation of adhesion molecules on the culture substrates or through an unidentified mechanism. We compared the effect of treatment with cathepsin G and other proteases, including neutrophil elastase against fibronectin- (FN-) coated substrates. Cathepsin G and elastase potently degraded FN on the substrates and induced aggregation of MCF-7 cells that had been subsequently seeded onto the substrate. However, substrate-bound cathepsin G and elastase may have caused cell aggregation. After inhibiting the proteases on the culture substrates using the irreversible inhibitor phenylmethylsulfonyl fluoride (PMSF), we examined whether aggregation of MCF-7 cells was suppressed. PMSF attenuated cell aggregation on cathepsin G-treated substrates, but the effect was weak in cells pretreated with high concentrations of cathepsin G. In contrast, PMSF did not suppress cell aggregation on elastase-treated FN. Moreover, cathepsin G, but not elastase, induced aggregation on poly-L-lysine substrates which are not decomposed by these enzymes, and the action of cathepsin G was nearly completely attenuated by PMSF. These results suggest that cathepsin G induces MCF-7 aggregation through a cell-oriented mechanism.

## 1. Introduction

Tumor cells in the tumor mass interact with adjacent tumor cells through homotypic adherence molecules such as E-cadherin on epithelial tumor cells. They also bind to the surrounding extracellular cell matrix (ECM) through integrins [1]. It is widely known that the process of cancer metastasis is accompanied by changes in the adherence capacity of tumor cells. For instance, the loss in the capacity for homotypic adherence, which is caused by downregulation of E-cadherin, is often observed in highly metastatic tumor cells. Loss of E-cadherin function is important in the acquisition of a more invasive phenotype to promote the dissemination of tumor cells from a tumor mass [1, 2]. In contrast, loss of integrin expression, which weakens cell-matrix interactions, reportedly correlates with the metastatic capacity of breast cancer cells. Additionally, it has been suggested that a reduction in the adherence capacity to the ECM induces formation of multicellular aggregates or spheroids of tumor cells, facilitating tumor cell dissemination [3–5]. The disseminated cell spheroids may cause emboli in blood vessels or lymph nodes [6–8]. Although changes in the activities of E-cadherin and integrins in tumor cells are important for tumor metastasis, the factors governing adherence capacity remain unknown.

Leukocytes, including neutrophils, infiltrate and accumulate in many tumors [9–11]. Neutrophils are thought to secrete a variety of factors, including proteases, cytotoxic factors, cytokines, and reactive oxygen species, that affect tumor growth and metastasis [12, 13]. These factors can have both beneficial and harmful effects on the host. To determine whether neutrophils produce factor(s) that alter(s) tumor cell adherence, we previously examined the effect of the neutrophil lysate on the adherence capacity of MCF-7 mammary breast carcinoma cells [14]. Serine proteases, cathepsin G, and neutrophil elastase (hereafter referred as to elastase) were shown to induce homotypic cell-cell aggregation* in vitro*. The molar activity of cathepsin G is stronger than that of elastase. The cell aggregates formed are E-cadherin-dependent three-dimensional (3D) multicellular spheroids [14, 15]. These observations indicate that neutrophil serine proteases affect tumor metastasis by modulating the adherence capacity of tumor cells. Cathepsin G may attenuate binding between integrin and extracellular adhesion molecules such as fibronectin (FN), resulting in E-cadherin-mediated homotypic cell-cell adhesion.

Cathepsin G is typically stored in the azurophilic granules of neutrophils and is released by a variety of stimuli, such as the presence of bacteria [16, 17]. Cathepsin G is not only involved in resistance against bacterial or fungal infection [17–20], but also has various physiological functions, such as induction of platelet activation [21, 22], induction of chemotaxis of leukocytes [23], activation of inflammatory cytokine release [24], induction of endothelium-dependent vascular relaxation [25], cardiomyocytes anoikis [26], and endothelial cell damage [27]. We previously reported that the aggregation-inducing activity of cathepsin G against MCF-7 cells is dependent on enzyme activity, while the binding of cathepsin G to specific binding sites on cells is independent of enzyme activity [28]. Accordingly, we hypothesized that cathepsin G binds to a specific protein on MCF-7 cells and causes proteolytic cleavage either of the binding protein itself or putative adjacent proteins in order to signal a change in adherent capacity. Cathepsin G is reportedly a ligand for protease-activated receptors (PARs) [17, 22, 24, 29]. PARs are G-protein coupled receptors in which cleavage of tethered ligands of receptor molecules by specific proteases is required for PAR activation. However, our previous study showed that agonist peptides for each PAR did not induce MCF-7 aggregation, suggesting that this function of cathepsin G may involve molecules other than PARs on the surface of MCF-7 cells.

It is well known that cathepsin G and elastase degrade adhesion molecules of the ECM such as FN [30–32]. Serum contains adherent molecules such as FN and vitronectin [33]; the presence of fetal bovine serum (FBS) in the culture medium is indispensable for the adherence of MCF-7 cells to culture substrates. Therefore, degradation of the adherence molecules by cathepsin G or elastase may reduce the adherence capacity of MCF-7 cells to adhesion molecules on culture substrates, inducing E-cadherin-dependent cell-cell adhesion, which is protease-resistant [34]. In this report, we examined whether neutrophil proteases, specifically cathepsin G and elastase, induce MCF-7 cell aggregation by degrading adhesion molecules on culture substrates or if a cell-oriented mechanism exists. For this, we determined the adherence capacity of cells when adhesion molecules on the culture substrates were treated with proteases prior to cell seeding. Cell aggregation occurred on the protease-treated substrate. To elucidate whether the aggregation observed on protease-treated substrates was solely due to the degradation of adhesion molecules on the culture substrates, protease-treated substrates were treated with phenylmethylsulfonyl fluoride (PMSF) or *α*_1_-antitrypsin, which are irreversible protease inhibitors, and cell aggregation was observed. For cathepsin G, the degree of cell adhesion was potently attenuated by treatment with protease inhibitors.

Among the proteases examined, the activity of cathepsin G induced MCF-7 cell aggregation, possibly involving a cell-oriented mechanism in addition to the degradation of adhesion molecules on the substrates. In contrast, induction of MCF-7 cell aggregation by elastase or other proteases, such as chymotrypsin and trypsin, appears to be dependent only on the degradation of adhesion molecules on culture substrates.

## 2. Materials and Methods

### 2.1. Reagents

Cathepsin G purified from human neutrophils (95% purity) was purchased from BioCentrum (Kraków, Poland). Bovine pancreas chymotrypsin (C7762) and bovine pancreas trypsin (T1426), *α*_1_-antitrypsin, PMSF, poly-L-lysine (PLL, MW; 150–300 kD), and fluorescein isothiocyanate (FITC)-labeled PLL (PLL-FITC, MWvis; 68.3 kD, 0.003–0.01 mol FITC per mol lysine monomer) were from Sigma (St. Louis, MO, USA). Elastase purified from human neutrophils and chymostatin were from Calbiochem (San Diego, CA). Human FN (FN, >95% purity) was from AGC Techno Glass (Chiba, Japan). Rabbit anti-human FN polyclonal antibody (AB1945) was obtained from Millipore (Billerica, MA, USA) and horseradish peroxidase- (HRP) conjugated secondary antibody was from Santa Cruz Biotechnology (Santa Cruz, CA, USA).

### 2.2. Cell Culture

The human breast cancer cell lines MCF-7 (E-cadherin-positive) and MDA-MB-231 (E-cadherin-negative) were used. MCF-7 cells were kindly provided by Dr. Hiroshi Kosano (Teikyo University, Japan) and MDA-MB-231 cells were obtained from ATCC (Manassas, VA, USA). Cells were maintained in RPMI 1640 medium supplemented with 10% heat-inactivated fetal bovine serum (FBS; MP Biomedicals, Solon, OH, USA) and 80 *μ*g/mL kanamycin (Wako Pure Chemical, Osaka, Japan) as described previously [28].

### 2.3. Measurement of Cell Motility

Cell motility was measured by chronological observation using a confocal laser microscope with a built-in CO_2_ incubator FV10i (OLYMPUS, Tokyo, Japan). Cells were inoculated on a glass-bottomed dish (AGC Techno Glass) in RPMI 1640 supplemented with 5% FBS on the day before the measurement. After changing the medium to the cathepsin G-containing RPMI 1640 supplemented with 1% bovine serum albumin (BSA) and NucRed Live 647 (Life Technologies, Carlsbad, CA, USA), which is a fluorescent probe used to visualize cell nuclei, cells were chronologically observed using confocal laser microscopy at 1-hour intervals. The incubator was maintained at 37°C in a humidified atmosphere of 5% CO_2_ in air. Cell motilities were quantified by drawing lines to connect between the center positions of the nuclei of the cell over 2 hours. The lines for 10 randomly selected cells in a visual field were quantified using software ImageJ.

### 2.4. Cell Aggregation Assays

To quantitatively assess the degree of cell aggregation or multicellular spheroid formation, we quantified the MCF-7 human breast cancer cells that were tightly attached to the culture plate after staining with crystal violet as previously described [28]. Briefly, MCF-7 cells (1 × 10^4^ cells/well) were seeded in 96-multiwell plates (without precoating; Iwaki Glass Inc., Tokyo, Japan) and cultured in RPMI 1640 medium containing 5% FBS for 24 h. After adherent cells were washed with serum-free RPMI 1640 medium, the medium was replaced with RPMI 1640 medium containing 1% BSA (1% BSA-medium) and cultivated with cathepsin G or other proteases, respectively. After cultivation for 24 h, the plate was vigorously tapped on paper towels 10 times to remove loosely attached cell aggregates and spheroids. Remaining cells were stained with 0.1% crystal violet in phosphate-buffered saline (PBS) for 10 min. After extensive washing with tap water, the plate was dried, and residual crystal violet in the cells was lysed with 100 *μ*L of 0.5% sodium dodecyl sulfate (SDS). Optical density at 595 nm (OD_595_) was measured, and the aggregation index was calculated as follows:

(1) Aggregation  index  (%) =OD595  without  sample−OD595  with  test  samplesOD595  without  sample  ×100.

Although some of the calculated aggregation index values were slightly below zero, microscopic observation revealed that the cells were morphologically similar to nonaggregated control cells, and, therefore, the negative values are expressed as zero in the figures to avoid confusion. The effect of proteases against MDA-MB-231 human breast cancer cells was also examined using the same assay. The effect of protease treatments on this cell type was expressed as the “detachment index” because cell aggregation did not occur.

### 2.5. Preparation of FBS-, FN-, or PLL-Coated Plates and Cell Aggregation on These Plates

To prepare FBS-coated plates, 96-multiwell plastic plates were incubated in 5% FBS-containing RPMI 1640 medium at 37°C overnight and were washed with serum-free medium before use. To prepare FN-coated plates, plates were treated with 10 *μ*g/mL FN in PBS at 4°C overnight, blocked in 3% BSA/PBS for 1 hour, washed once with serum-free medium, and immediately used. For PLL-coating, the multiwell plates were incubated with PLL solution (0.01%, w/v in PBS) at 37°C for 1 hour, washed with sterile purified water three times, dried, and rewashed once with serum-free medium before use.

To examine the effect of protease treatment on cell aggregation in FBS-, FN-, or PLL-coated plates, proteases in 1% BSA-medium were added to the coated plates, and the plates were incubated at 37°C for 24 hours. Plates were then washed once with serum-free medium and MCF-7 cells (1 × 10^4^ cells/well) were seeded. After 24 hours of culture, the aggregation index was evaluated as described above. In some experiments, to inhibit proteases bound to the wells, the irreversible protease inhibitors PMSF or *α*_1_-antitrypsin were used. Briefly, 1% BSA-medium solution containing 4 mM PMSF or 200 *μ*g/mL *α*_1_-antitrypsin was added to protease-treated plates, which were incubated at 37°C for 1 hour. For PMSF, 1% dimethyl sulfoxide (DMSO) in 1% BSA-medium was used as a vehicle control. After the treatment with protease inhibitors, the plates were washed once with serum-free medium before adding the cell suspension.

### 2.6. Measurement of Cathepsin G and Elastase Enzymatic Activities

The enzymatic activity of cathepsin G bound to the culture plates was measured using N-succinyl-Ala-Ala-Pro-Phe* p*-nitroanilide (Sigma) as a substrate [35]; 200 *μ*L of the substrate (1.2 mg/mL) in HEPES buffer (pH 7.5) containing 10% DMSO was added to the wells and incubated at 37°C for 90 min. After incubation, liberated* p*-nitroanilide was evaluated by measuring changes in the optical density at 405 nm (OD_405_). To measure elastase in the wells, elastase substrate I (Calbiochem) was used as a substrate, and the levels of liberated* p*-nitroanilide were measured after 10 min.

### 2.7. Western Blotting Analysis of FN

The amount of FN that remained adhered to the culture wells after protease treatment was analyzed by western blotting. Each protease-treated culture well was washed once with PBS, after which 20 *μ*L Laemmli's sample buffer containing 2-mercaptoethanol was added. After extensive scraping of the wells with micropipette tips, the solutions from three wells were pooled, and the samples were separated by SDS-PAGE. Western blotting was performed using an anti-FN rabbit polyclonal antibody and an HRP-labeled secondary antibody as previously described [28].

### 2.8. Cleavage Activity of the Proteases towards PLL-FITC

PLL-FITC (100 *μ*g/mL in PBS) was mixed with each protease, and the 1 mL reaction mixtures were incubated at 37°C for 4 hours. Next, the solutions were applied to a Millipore ultrafiltration system (membrane cut-off of 10 kDa) and the fractions (<10,000 fr.) that passed through the ultrafiltration unit were diluted 10-fold with PBS. Each 200 *μ*L aliquot was added into a 96-well microplate (Nunclon DELTA Surface, Nunc, Roskilde, Denmark), and the fluorescence intensity of FITC was measured using a multimode plate reader (485 nm excitation wavelength, 535 nm emission wavelength; ARVO X3, PerkinElmer, Waltham, MA, USA).

### 2.9. Statistical Analysis

For statistical analysis of the data, Student's* t*-tests were used. Data are expressed as the mean ± standard deviation (SD), unless otherwise indicated.

## 3. Results 

### 3.1. Augmentation of Cell Motility by Cathepsin G

**Figure 1:**
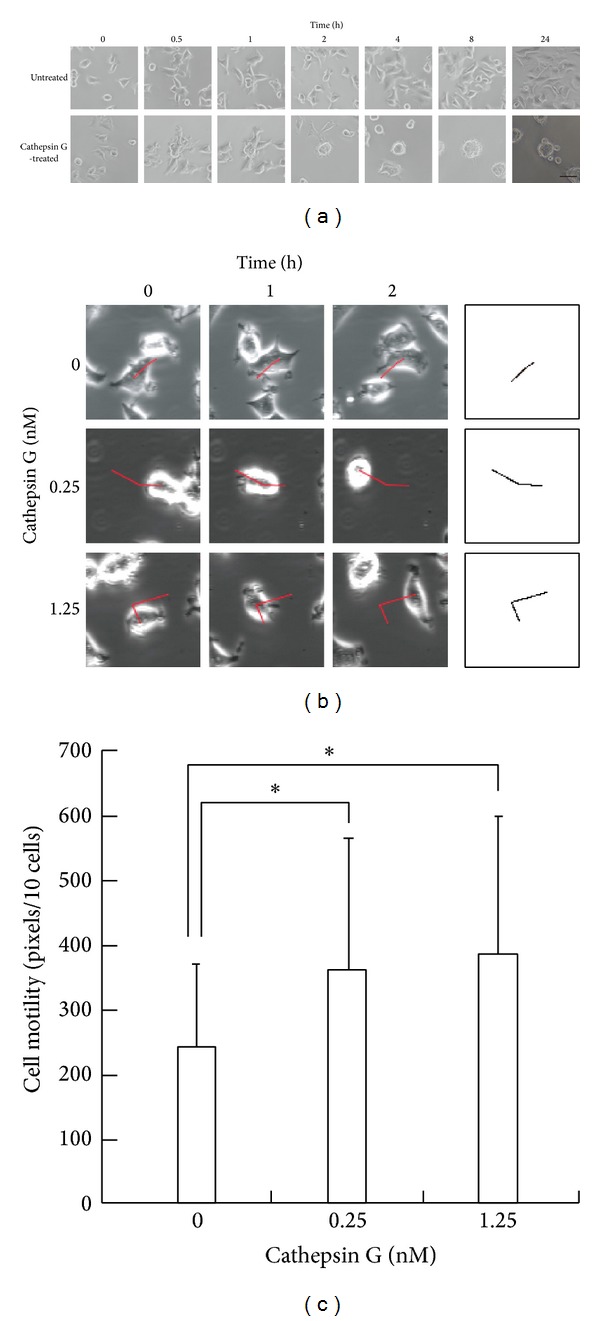
Cathepsin G increases the motility of MCF-7 cells. (a) Time course of the formation of MCF-7 cells aggregates by cathepsin G. MCF-7 cells (1 × 10^4^ cells/well) were seeded in a 96-multiwell plate in RPMI 1640 medium containing 5% FBS. The cells were cultured for 24 h and then incubated without or with cathepsin G (40 nM) in medium containing 1% BSA for the indicated periods. Scale bar = 50 *μ*m. (b) Quantification of cathepsin G-treated MCF-7 cell motility. The cells were grown on a glass-based dish in RPMI 1640 supplemented with 5% FBS the day before measurements were taken. After changing the medium to RPMI 1640 supplemented with 1% BSA, the cells were chronologically observed using confocal laser microscopy with a built-in CO_2_ incubator at 37°C in a humidified atmosphere of 5% CO_2_ in air. The trajectories of the cells are shown (red lines in left panels and black lines in right panels). (c) Cell motility of cathepsin G-treated MCF-7 cells. The trajectories of the 10 cells that were randomly selected in a visual field were quantified using imaging software ImageJ. The results are shown as means ± SD (*n* = 5). Asterisk indicates that the values are significantly different according to the Student's* t*-test (*P* < 0.05).

We previously observed that when cathepsin G was added to adherent MCF-7 cells, the cells moved to contact each other and form cell aggregates, eventually forming 3D-sheroidal shapes when adherence to substrates is reduced. To characterize the early phase of this reaction, we first quantified the degree of cell movement when cells were cultured with cathepsin G.  Figure 1(a) shows that only in the presence of cathepsin G did the cells generally touch each other at 0.5 hours and maintain their cell-cell adhesions during subsequent culturing, finally forming spheroids. Quantitative analysis was performed with MCF-7 cells cultured on a glass-based dish at lower concentration (0.25 or 1.25 nM) of cathepsin G (Figures 1(b) and 1(c)). Notably, MCF-7 cells cultured on a glass-based dish (Figure 1(b)) were more sensitive to cathepsin G than cells cultured on a plastic plate (Figure 1(a)), presumably because adhesion properties are weaker on a glass-based dish. In fact, MCF-7 cells treated with 40 nM cathepsin G formed complete spheroids on a glass-based dish even after 1 hour (data not shown), although the cathepsin G-treated cells completely formed spheroids on a plastic plate after 4 hours (Figure 1(a)). The results showed that the degree of cell movement was significantly augmented by cathepsin G, indicating that cathepsin G actively induced spheroidal aggregate formation of MCF-7 cells by altering cell motility (Figures 1(b) and 1(c)). In contrast, elastase (0.25–10 nM) did not increase the motility of the cells (see Supplementary Figure 1 in Supplementary Materials available online at http://dx.doi.org/10.1155/2014/971409).

### 3.2. Comparison of the Effects of Serine Proteases on the Adherence Capacity of MCF-7 and MDA-MB-231 Cells

**Figure 2:**
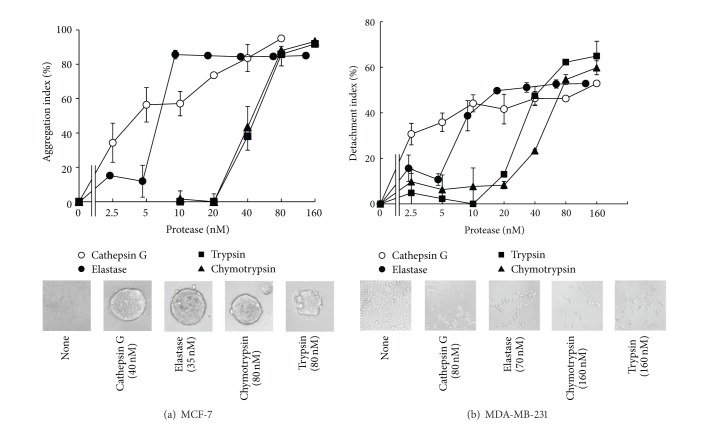
Effect of serine proteases on the adherence capacity of preadhered mammary carcinoma cells.(a) Induction of spheroidal aggregation by proteases against MCF-7 cells. (b) Induction of the loss of tight adhesion to culture substrates by serine proteases against MDA MB-231 cells. ((a), (b)) MCF-7 cells or MDA MB-231 cells were seeded in 96-well plates in RPMI 1640 medium containing 5% FBS. The cells were cultured overnight and the adherent cells were incubated with cathepsin G (○), neutrophil elastase (●), chymotrypsin (▲), or trypsin (■) containing 1% BSA-medium for 24 h. After washing, residual cells were stained with crystal violet, and the aggregation index (a) or the detachment index (b) was calculated as described in Section 2. The results are expressed as mean ± SD (*n* = 3). When the bars are not shown, they are smaller than the size of the symbols. Lower photos: images of MCF-7 cells (left) and MDA MB-231 cells (right) at 24 h after incubation with the serine proteases. Original magnification: ×100.

Cathepsin G shows more potent aggregation-inducing activity against MCF-7 cells than does elastase [14] or chymotrypsin [28]. Since cathepsin G has chymotrypsin-like and trypsin-like substrate specificities, we first compared the activity of cathepsin G with elastase, chymotrypsin, and trypsin. Cathepsin G induced MCF-7 aggregation at low concentrations; cathepsin G induced aggregation in a dose-dependent manner beginning at a concentration of 2.5 nM, while the aggregation-inducing activity of elastase was observed beginning at approximately 10 nM in an all-or-none manner (Figure 2(a)). In contrast, higher concentrations (greater than 40 nM) of chymotrypsin and trypsin were required to induce aggregation. Thus, among these proteases, cathepsin G was the most potent inducer of MCF-7 spheroidal aggregation.

Since aggregation induction of MCF-7 cells by cathepsin G is E-cadherin-dependent [14, 15], we next examined how these proteases affect the morphology of E-cadherin-negative MDA-MB-231 cells. When left overnight in the presence of each protease, the cells lost the spindle morphology and detached easily during the washing step of crystal violet staining (Figure 2(b)). However, in contrast to the cadherin-positive MCF-7 cells, aggregation of MDA-MB-231 cells was not induced by any of the proteases. The dose-response relationships of these proteases in inducing the detachment of MDA-MB-231 cells were similar to those that induced aggregation of MCF-7 cells; the effects of cathepsin G and elastase were observed beginning at concentrations of 2.5 nM and 10 nM, respectively, while the effects of chymotrypsin and trypsin were observed beginning at 40 nM (Figure 2(a)). These findings suggest that these proteases affect interactions between cells and culture substrates and that these changes occur regardless of E-cadherin expression.

### 3.3. Effect of Treatment with Each Protease against Culture Substrates Coated with Adhesion Molecules

**Figure 3:**
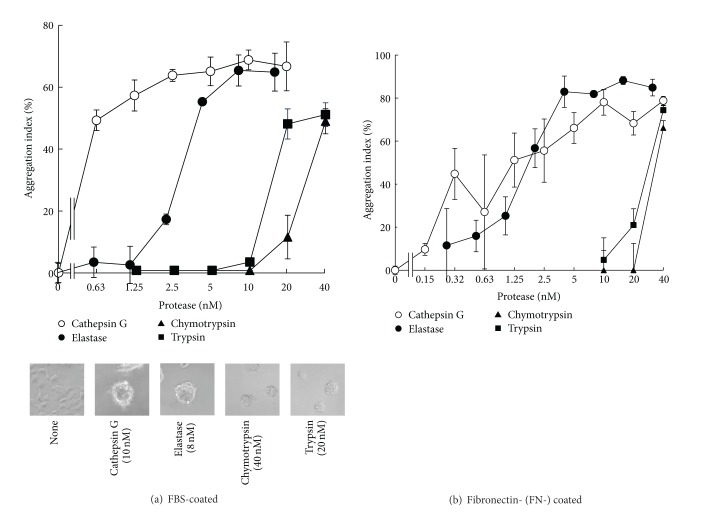
Induction of MCF-7 cell aggregation by serine protease treatment of FBS- or FN-coated wells. FBS-coated wells (a) or FN-coated wells (b) were treated overnight with cathepsin G (○), elastase (●), chymotrypsin (▲), or trypsin (■). Next, MCF-7 cells suspended in 1% BSA-medium were seeded and incubated for 24 h. The cell aggregation index was evaluated as described in the legend of Figure 2. The results are expressed as means ± SD (*n* = 3). When the bars are not shown, they are smaller than the size of the symbols. Lower photos: images of MCF-7 cells at 24 h after cultivation in protease-treated FBS-coated wells. Original magnification: ×100.

Evaluation of the adherence of MCF-7 cells to the culture substrate requires the presence of adhesion molecules in the substrates because the cells only adhere to plastic plates when FBS is in the medium. In the experiments shown in Figure 2, culture plates were not precoated with adhesion molecules before cell seeding; it was thought that adhesion factors such as FN and vitronectin in FBS became bound to the plates during the preculture of MCF-7 cells, maintaining the adhesion of these cells. To understand the implication of adhesion molecule degradation by proteases and the reduction of substrate adherence of MCF-7 cells, we examined cell aggregation on FBS-coated wells that had been treated overnight with each protease prior to adding cells. As shown in Figure 3(a), cell aggregation was observed with all protease-treated substrates. The effect of cathepsin G pretreatment was clear at lower concentration ranges compared to that of elastase, and chymotrypsin and trypsin required much higher concentrations to take effect than cathepsin G and elastase. Thus, overall dose-response relationships showed similar trends as those shown in Figure 2(a), which reflect results obtained when the proteases were added to adhered MCF-7 cells.

Because FBS contains multiple adhesion molecules, including FN and vitronectin, as well as inhibitory factors such as *α*_1_-antitrypsin and *α*_1_-antichymotrypsin against serine proteases, we performed similar experiments using human FN rather than FBS to coat the culture substrates.  Figure 3(b) shows that protease treatments of FN-coated plates induced aggregation of MCF-7 cells with a dose-response relationship similar to that observed for FBS-coated plates. Cathepsin G morphologically induced cell aggregation even at 0.32~0.63 ng/mL (not shown), although the deviation of the aggregation index at 0.63 ng/mL was very high. The effect of elastase on FN-coated plates was observed at lower concentration ranges than that on FBS-coated plates. The reason for this is unclear, but elastase might exert more potent effect in FN-coated plates due to the lack of the FBS-derived protease inhibitors.

**Figure 4:**
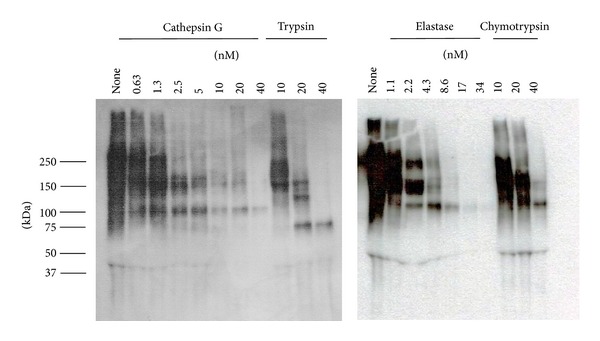
Residual amounts of FN on the bottoms of FN-coated wells that had been treated overnight with each protease. FN bound to the bottoms was collected after a washing and analyzed by western blotting as described in Section 2.

To examine whether the extent of cell aggregation on protease-treated FN was correlated with the amounts of FN remaining in the culture after treatment, residual FN was estimated by western blotting. As shown in Figure 4, cathepsin G and elastase digested FN (molecular weight under reduced conditions was 220 kD) much more effectively than trypsin and chymotrypsin; a reduction in residual FN was observed from 2.5 nM cathepsin G and elastase and from 20 nM of chymotrypsin and trypsin. At lower concentrations of cathepsin G (0.63 and 1.25 nM), some FN remained. However, cell aggregation was induced by cathepsin G at 1.25 nM and below (Figure 3(b)), indicating that cathepsin G induces cell aggregation through an additional mechanism other than substrate adhesion molecule degradation. In contrast, elastase caused potent decomposition of FN at about 2 nM, and it was entirely decomposed at approximately 5 nM. Since the potent cell aggregation induced by elastase at approximately 2 nM plateaued when concentrations reached 5 nM, the increase in the aggregation index was correlated with the degree of FN degradation.

For trypsin and chymotrypsin, cell aggregation was observed at around 40 nM (Figure 3(b)); at this concentration, FN was almost entirely decomposed. At 20 nM of concentration for both enzymes, cell aggregation was either weakly induced or not induced at all (Figure 3(b)), although extensive FN decomposition was observed. It is likely that the residual FN fragment may have been able to sustain cell adherence, although further experiments are necessary to determine the validity of this hypothesis.

### 3.4. Inhibition of Cathepsin G Protease Activity Remaining in Culture Substrates by PMSF Attenuated the Induction of MCF-7 Cell Aggregation

To determine whether cathepsin G or elastase induces cell aggregation when being bound to culture substrates, we next measured the enzymatic activities remaining in the culture substrates after FN-coated plates were treated with proteases and washed out. Supplementary Figure 2 shows that approximately one-third of the enzymatic activities of the added cathepsin G and elastase remained. Therefore, cathepsin G or elastase bound to the bottom of the wells may induce cell aggregation through their enzymatic activity. Consequently, we examined whether cell aggregation on the cathepsin G- or elastase-treated FN-coated wells is attenuated by treatment with PMSF against substrate-bound enzymes. If the cell aggregation was induced by adhesion molecule digestion on the culture substrates by the proteases, aggregation intensity should not be influenced by PMSF treatment.

**Figure 5:**
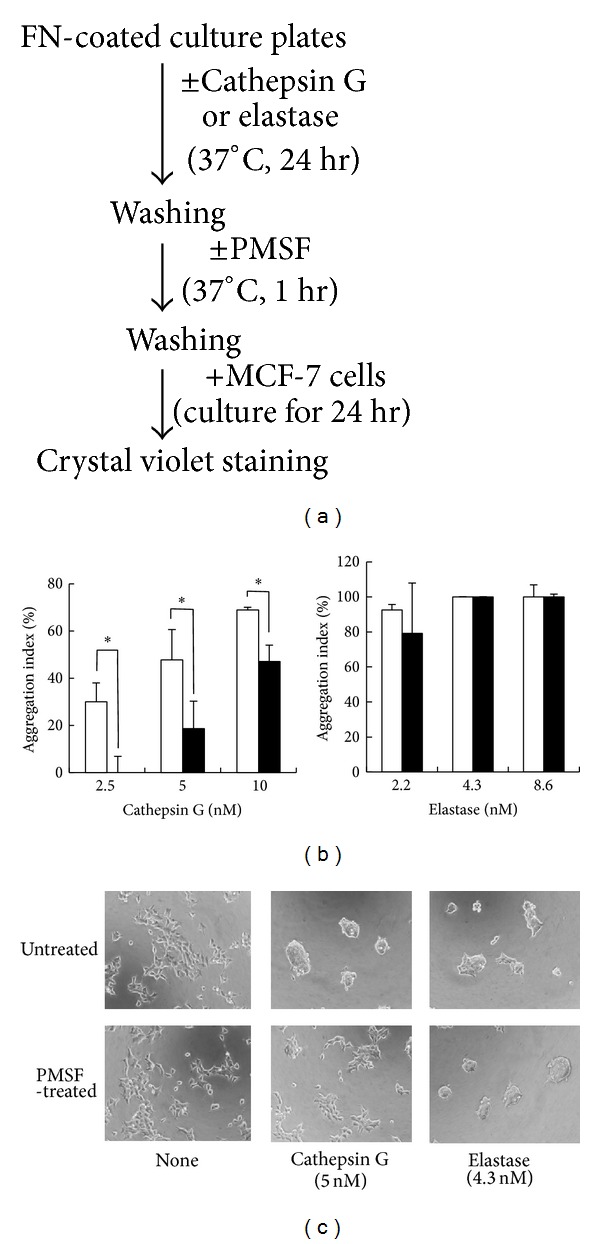
Effect of PMSF treatment against protease-treated FN-coated wells on the induction of MCF-7 cell aggregation. (a) Experimental scheme. Cathepsin G or elastase was added to FN-coated wells as indicated. After 24 h of incubation, the wells were treated without or with PMSF (4 mM) at 37°C for 1 h. Then, MCF-7 cells suspended in 1% BSA-medium were seeded and incubated for 24 h. The cell aggregation index was evaluated as described in the legend of Figure 2. (b) Open columns: without PMSF treatment (vehicle control); filled columns: with PMSF treatment. The results are expressed as the mean ± SD (*n* = 3). Asterisk indicates that the values are significantly different according to the Student's* t*-test (*P* < 0.05). (c) Images of MCF-7 cells at 24 h after cultivation in protease/PMSF-treated FN-coated wells. Original magnification: ×100.

The enzymatic activities of cathepsin G and elastase in the bottom of the plate wells were nearly completely or extensively inhibited by PMSF treatment (Supplementary Figure 2), suggesting that PMSF is effective against proteases bound to the culture substrates. As shown in Figures 5(b) and 5(c), the cell aggregation-inducing effect of 2.5 and 5 nM cathepsin G treatments against FN-precoated plates were markedly attenuated by subsequent treatment with PMSF, while the aggregation caused by 10 nM treatment of cathepsin G was suppressed by PMSF to a lesser degree. In contrast, the aggregation-inhibiting effects of elastase (2.2–8.6 nM) were not disturbed by PMSF. These results suggest that cathepsin G induces cell aggregation by not only degrading adhesion molecules, but also through putative cell-oriented mechanisms, and that at the higher concentration (10 nM), extensive decomposition of FN on the substrates is the primary cause of aggregation. In contrast, elastase may induce cell aggregation solely by degrading FN.

Similar results were obtained using FBS-coated plates rather than FN-precoated plates (Supplementary Figure 3); the cell aggregation-inducing activity of treatment with 0.63 nM cathepsin G against FBS-precoated plates was completely suppressed by subsequent treatment with PMSF, whereas those of 2.5 and 10 nM cathepsin G were only weakly reduced. The effects of elastase, chymotrypsin, and trypsin treatments were unaffected by PMSF. Similar results were also obtained using *α*_1_-antitrypsin rather than PMSF as an irreversible protease inhibitor (data not shown). These results support the notion that cathepsin G induces cell aggregation through a cell-oriented mechanism that is dependent on enzymatic activity. However, at higher concentrations, the degradation effect of cathepsin G against FN may mask cell-orientation activity.

To further support that MCF-7 cell aggregation by cathepsin G is not solely caused by the degradation of adhesion molecules, we next examined the effect of the proteases on poly-L-lysine- (PLL-) coated plates because PLL was not expected to be a good substrate for cathepsin G. To measure the hydrolytic activity of each protease towards PLL, we mixed PLL-FITC with each protease for 37°C for 1 hour and then measured FITC fluorescence in fractions that had been ultrafiltered (10 kD cut-off). Supplementary Figure 4 shows that cathepsin G and elastase treatments did not augment fluorescence in fractions <10 kD, whereas chymotrypsin and trypsin treatment revealed small and large increases, respectively. The fluorescence intensity of degraded PLL-FITC may be higher than that of the intact molecule because fluorescence of the <10 kD fraction of the trypsin-treated PLL-FITC was higher than that of the untreated control. These results indicate that, in contrast to chymotrypsin and trypsin, cathepsin G and elastase show no cleavage activity towards PLL. Additionally, we examined cell aggregation in PLL-coated plates that had been treated with cathepsin G and elastase.

**Figure 6:**
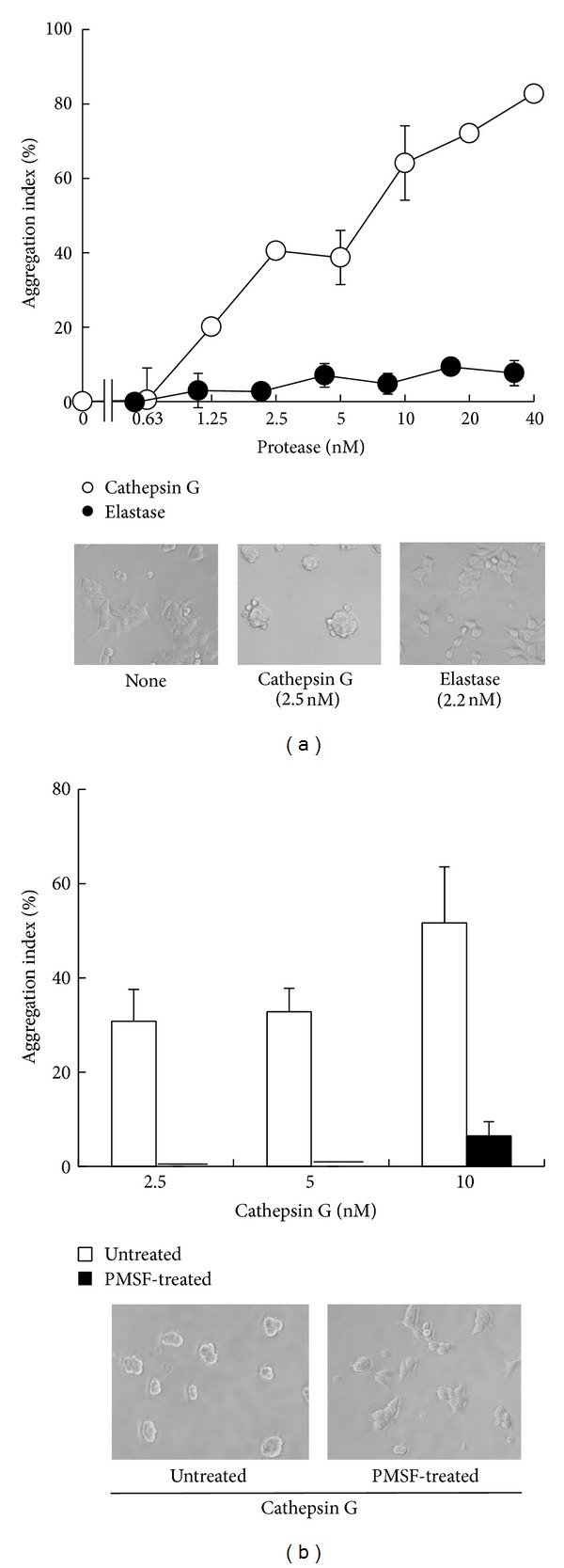
Induction of MCF-7 cell aggregation by cathepsin G, but not elastase, treatment of PLL-coated wells. (a) PLL-coated wells were treated overnight with cathepsin G (○) or elastase (●). After the enzymes were removed by washing, MCF-7 cells suspended in 1% BSA-medium were seeded and incubated for an additional 24 h. The cell aggregation index was evaluated as described in the legend of Figure 2. The results are expressed as the mean ± SD (*n* = 3). When the bars are not shown, they are smaller than the size of the symbols. Original magnification: ×100. (b) PLL-coated wells were treated overnight with cathepsin G for 24 h, and then the wells were washed and treated without (open column) or with PMSF (4 mM, filled column) at 37°C for 1 h. MCF-7 cells suspended in 1% BSA-medium were then seeded and incubated for an additional 24 h. Cell aggregation was evaluated as above. Lower photos: images of MCF-7 cells at 24 h after cultivation in the cathepsin G (2.5 nM)-treated PLL-coated wells, subsequently treated without (left) or with (right) PMSF.

Figure 6(a) shows that cathepsin G treatment against the PLL substrate induced MCF-7 cell aggregation, whereas elastase pretreatment did not induce aggregation. We confirmed that the amounts of cathepsin G bound to the bottoms of the PLL-precoated plates were similar to those bound to FN-coated plates and that the activity of substrate-bound cathepsin G was nearly completely inhibited by PMSF treatment (data not shown).  Figure 6(b) shows that cell aggregation was not observed when substrate-bound cathepsin G was inhibited by PMSF treatment prior to cell seeding. These data support that cathepsin G, but not elastase, induces cell aggregation through an enzyme activity-dependent, cell-oriented mechanism.

## 4. Discussion

In this study, we found that cathepsin G induces the formation of multicellular aggregates in low metastatic and E-cadherin-positive MCF-7 cells, while reducing the adherent capacity of highly metastatic and E-cadherin-negative MDA MB-231 cells to culture substrates. Because the dose-response curves of cathepsin G inducing changes in the adherence capacity of MCF-7 and MDA MB-231 cells are very similar, cathepsin G may have a common molecular mechanism in the induction of reduced cell-substrate interactions among these two cell types. We previously reported that cathepsin G-induced cell aggregation is E-cadherin-dependent [14, 15]. This hypothesis was supported in the current study because cathepsin G did not induce aggregation of E-cadherin-negative MDA MB-231 cells.

Two mechanisms have been proposed for the dissemination mechanism of tumor cells from the tumor mass. First, cancer cells gain the capacity to infiltrate the ECM by augmentation of the integrin-mediated adhesive activity to adhesion molecules in the ECM while also increasing the expression of metalloproteinases. Such highly metastatic cells reportedly lose cadherin-dependent adhesive activity between tumor cells [1, 2]. Second, it was proposed that tumor cells lose the capacity to adhere to the ECM [3–5] and that they form homotypic aggregates, disseminate into small vessels, and become trapped as tumor emboli [6–8]. Cathepsin G secreted by neutrophils infiltrating intratumor environments is a candidate factor that influences the metastatic capacity of tumor cells. Cathepsin G may act on E-cadherin-positive tumor cells to induce detachment from the ECM to facilitate the dissemination of tumor cell aggregates. In contrast, cathepsin G-caused suppression of the adhesion capacity between highly metastatic E-cadherin-negative tumor cells and the ECM may inhibit metastasis. However, future studies are needed to examine these possibilities.

We observed that in the presence of cathepsin G, MCF-7 cells move to make contact with adjacent cells. The quantification assay of cell motility showed that cathepsin G augments the motility of MCF-7 cells, suggesting that cell aggregation is not passively caused by the loss of adhesion to the substrates, but is caused by increased cell motility. In addition to MCF-7 cells, a comparison to other proteases of the effect of cathepsin G on the motility of other tumor cells, including highly metastatic MDA MB-231 cells, should be further examined. On the other hand, elastase did not increase the motility, suggesting that the reaction mode of cathepsin G in induction of cellular spheroids is different from that of elastase.

The aim of the present study was to determine whether the induction of cell aggregation of cathepsin G and other proteases is cell-oriented or is the result of adhesion molecule degradation on the culture substrates. We first examined the effect of protease treatment on a culture substrate precoated with FBS or FN. Protease treatments against FBS-coated plates induced cell aggregation of MCF-7 cells with a similar overall dose-response relationship as the results obtained when the proteases were added to adherent cells. Cathepsin G induced aggregation at the lowest concentration ranges in both assay systems, elastase exerted its effect at higher concentrations than cathepsin G, and chymotrypsin and trypsin only induced the aggregation at much higher concentrations. A similar order of the dose-response relationship was observed when FN-coated plates were treated with proteases. Since 10^6^ neutrophils reportedly secrete approximately 1 *μ*g of cathepsin G [36] and the concentration of cathepsin G that induces the cell aggregation is less than 40 nM (1 *μ*g/mL), the concentrations are physiologically relevant in environments containing relatively low amounts of inhibitory factors, such as serpines.

To understand whether the activity of cathepsin G and other proteases depends only on the digestion of adhesion molecules on the culture substrate, we used western blotting analysis to first estimate FN decomposition after cathepsin G treatment of FN-coated plates. In contrast to other proteases examined, cathepsin G induced cell aggregation at low concentrations, at which a substantial amount of FN remained. Therefore, cathepsin G may exert its effect through a mechanism other than digestion of adhesion molecules. This hypothesis was supported by the observation that PMSF attenuated the degree of cell aggregation on cathepsin G-treated FN plates. The inhibitory activity of PMSF against aggregation was clearly observed at relatively low concentrations (2.5–5 nM) of cathepsin G. In contrast, the effect of PMSF against 10 nM cathepsin G-treated FN plates was very weak, suggesting that cathepsin G-induced degradation of FN induces cell aggregation at higher concentrations. Accordingly, cathepsin G bound to the culture substrates may exert cell-oriented cell aggregation-inducing activity; however, the cell-oriented activity may be masked by the degradation effect of cathepsin G against FN at higher concentrations of cathepsin G. In contrast to cathepsin G, the activities of elastase, chymotrypsin, and trypsin appeared to depend only on adhesion molecule degradation on the culture substrates because PMSF did not suppress the induction of cell aggregation in FN- or FBS-coated plates treated with any of these enzymes.

Treatment with cathepsin G, but not elastase, induced cell aggregation on PLL substrates, although cathepsin G did not decompose PLL. Moreover, the activity of cathepsin G on PLL was nearly completely inhibited by subsequent PMSF treatment. These results strongly support that cathepsin G affects the character of cell adherence of MCF-7 through a possible cell-oriented mechanism, although this mechanism remains unknown. We previously reported that cathepsin G binds to a specific binding site on MCF-7 cells [28]. Identifying this binding site is key to resolving the mechanism of cell aggregation induction. Recently, Zen et al. reported that the neutrophil serine proteases cathepsin G, elastase, and proteinase-3 cleave *β*_2_ integrin CD11b/CD18 to inhibit neutrophil chemotaxis [37], although another report indicated that cathepsin G modulates integrin CD11b clustering without proteolytical procession of integrin molecules [38]. One possibility is that cathepsin G alters the character of adherence by cleaving the integrins of tumor cells. The results of this study are that cathepsin G induced cell aggregation not only on FN-coated substrates but also on PLL-coated surfaces and cell adhesion to PLL is not dependent on integrins. Because cathepsin G loses extended MCF-7 morphology, it is thought that cathepsin G decreases focal adhesion of the cells. We are planning to examine whether cathepsin G modulates formation of focal adhesion complex.

In summary, in contrast to elastase, cathepsin G may induce cell aggregation of MCF-7 cells through a cell-oriented mechanism in addition to degrading substrate adhesion molecules. Further studies are needed to identify the binding molecule(s) of cathepsin G in cells to elucidate the mechanism underlining this nonoverlapping activity of cathepsin G-induced alterations in the adherence capacity of tumor cells. These results contribute to the understanding of tumor invasion and metastasis and the role of neutrophils.

## Supplementary Material

Supplementary figure 1: Elastase does not increase MCF-7 cell motility.
Supplementary figure 2: The enzymatic activity of cathepsin G and Elastase bound to the bottoms of FN-coated plates.
Supplementary figure 3: Effect of PMSF treatment against protease-treated FPS-coated wells on the induction of MCF-7 cell aggregation.
Supplementary figure 4: PLL-FITC was not a substrate for cathepsin G and Elastase.
