# Supplementary material for: Neutrophil Cathepsin G, but Not Elastase, Induces Aggregation of MCF-7 Mammary Carcinoma Cells by a Protease Activity-Dependent Cell-Oriented Mechanism
Source: Mediators Inflamm. 2014 Apr 2;2014:971409. doi: 10.1155/2014/971409 (PMC3996324; doi:10.1155/2014/971409)

Supplementary fig. 1 Elastase does not increase MCF-7 cell motility. The cells were grown on a glass-based dish in RPMI 1640 supplemented with 5% FBS the day before measurements were taken. After changing the medium to RPMI 1640 supplemented with 1% BSA, the cells were added with elastase and were chronologically observed using confocal laser microscopy with a built-in CO2 incubator at 37°C in a humidified atmosphere of 5% CO2 in air. Cell motility was quantified by drawing lines to connect between the center positions of the nuclei of the cell over 2 hours. The trajectories of the 10 cells that were randomly selected in a visual field were quantified using imaging software ImageJ. The results are shown as means ± SD (n = 5). No significant differences are observed between the reference control and each elastase-treated group.

Supplementary fig. 2 The enzymatic activity of cathepsin G (A) and elastase (B) bound to the bottoms of FN-coated plates. FN-coated plates were incubated with 40 nM cathepsin G or 20 nM elastase at 37°C overnight without or with an additional 1-h treatment with 4 mM PMSF. After washing, the respective residual enzymatic activities were measured as described in Section 2. Simultaneously, the activity of the identical amount of each protease used per well for the treatment of FN-coated plates was measured and expressed as "Whole". The results are expressed as the mean ± SD (n = 3).

Supplementary fig. 3 Effect of PMSF treatment against protease-treated FBS-coated wells on the induction of MCF-7 cell aggregation. (A) Experimental scheme. FBS-coated wells were incubated with each serine protease for 24 h. Next, the wells were treated without or with PMSF (4 mM), following which MCF-7 cells were added, and the cell aggregation index was evaluated as described in the legend of Figure 5. (B) Open columns: without PMSF treatment (vehicle control), filled columns: with PMSF treatment. The results are expressed as the mean ± SD (n = 3). Asterisk indicates that the values are significantly different according to the Student's *t*-test ($p < 0.05$).

Supplementary fig. 4 PLL-FITC was not a substrate for cathepsin G and elastase. PLL-FITC was treated with the respective protease (40 nM cathepsin G, 35 nM elastase, 40 nM chymotrypsin, 40 nM trypsin) at 37°C for 4 h. After incubation, the reaction mixtures were passed through an ultrafiltration membrane, and fluorescence of FITC in the < 10 kD fraction was measured and compared with that of the untreated PLL-FITC, as described in Section 2. "Whole" represents untreated and unfractionated PLL-FITC.

Supplementary fig. 1

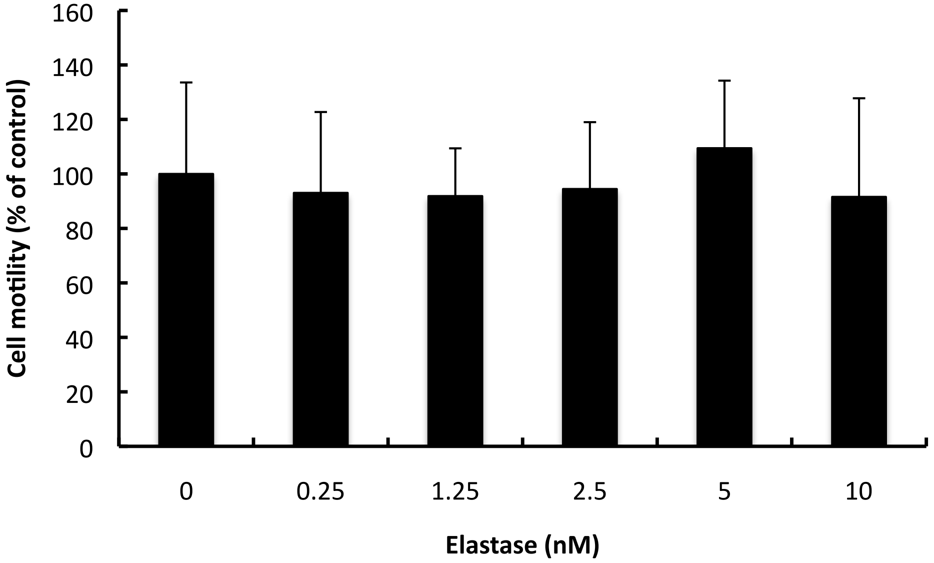

Supplementary fig. 2

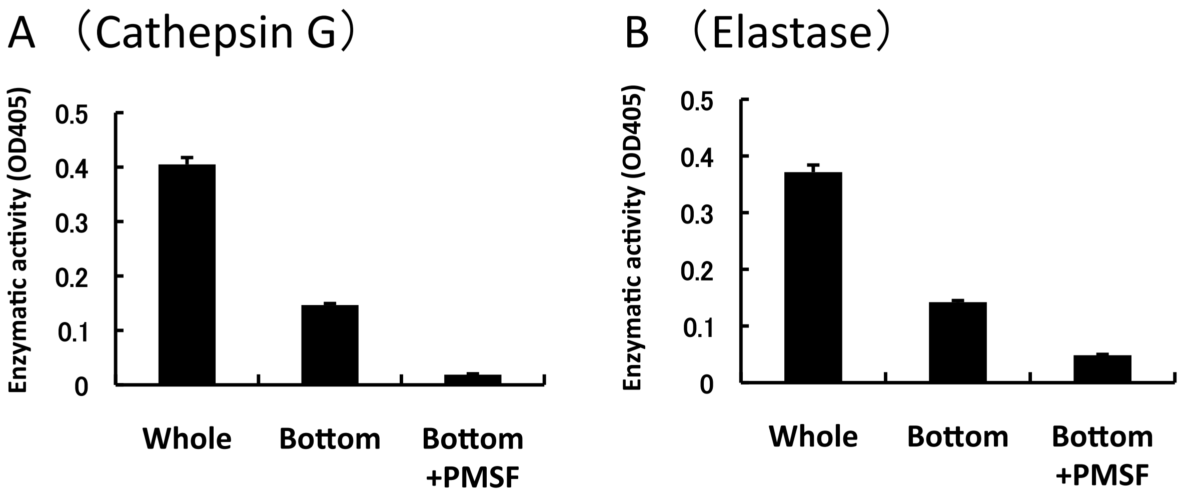

Supplementary fig. 3

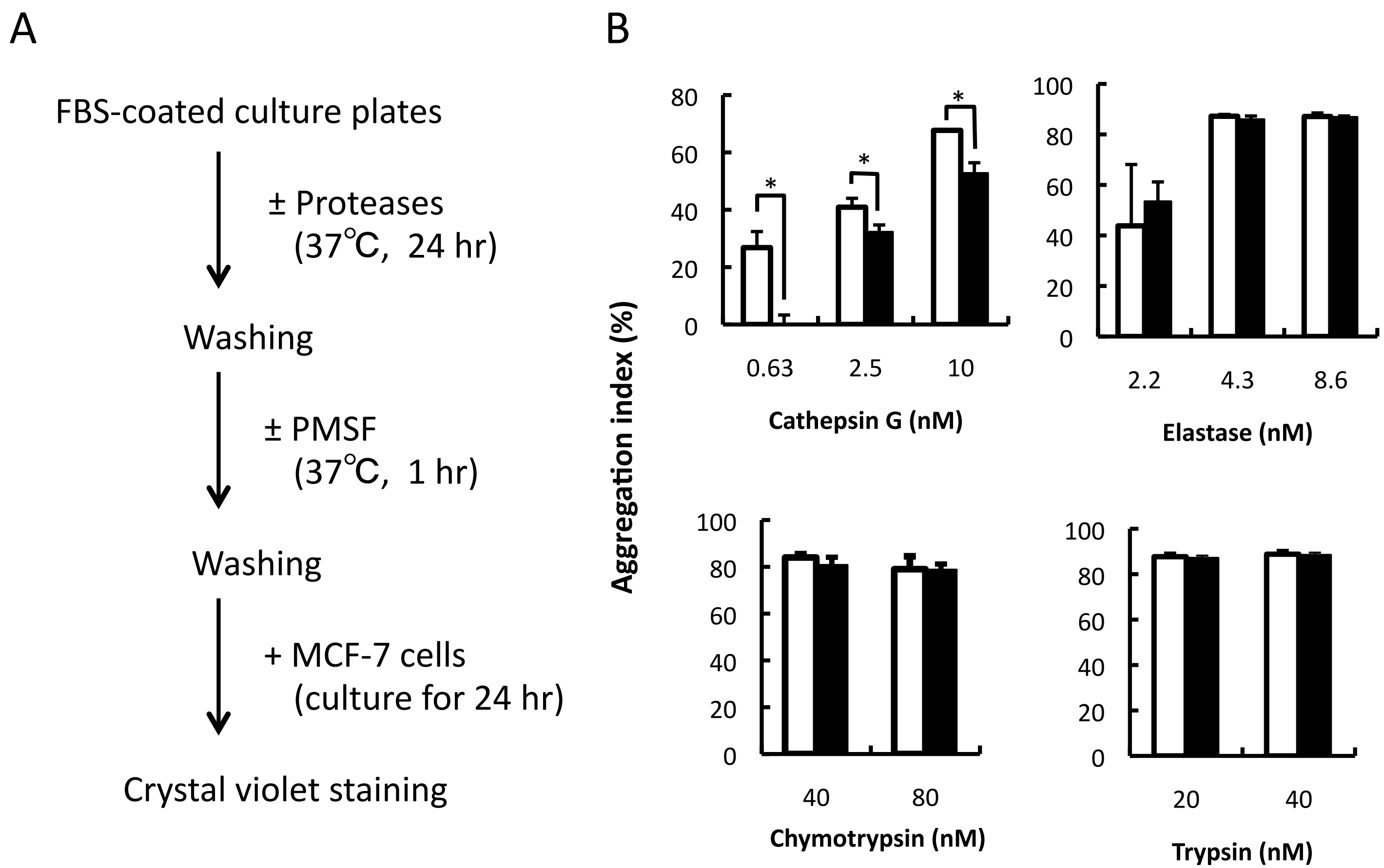

Supplementary fig. 4

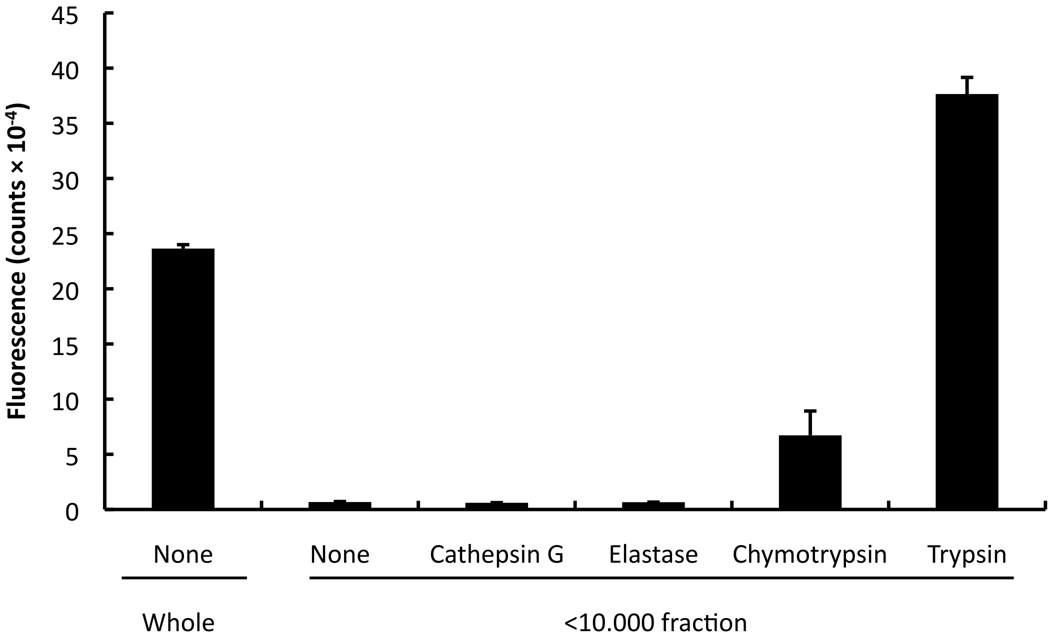

Supplement: Supplementary file 1 — Supplementary figure 1: Elastase does not increase MCF-7 cell motility. Supplementary figure 2: The enzymatic activity of cathepsin G and Elastase bound to the bottoms of FN-coated plates. Supplementary figure 3: Effect of PMSF treatment against protease-treated FPS-coated wells on the induction of MCF-7 cell aggregation. Supplementary figure 4: PLL-FITC was not a substrate for cathepsin G and Elastase. [file 971409.f1.pdf]
